# Disparities in COVID-19 Incidence, Hospitalizations, and Testing, by Area-Level Deprivation — Utah, March 3–July 9, 2020

**DOI:** 10.15585/mmwr.mm6938a4

**Published:** 2020-09-25

**Authors:** Nathaniel M. Lewis, Mike Friedrichs, Shelly Wagstaff, Kylie Sage, Nathan LaCross, David Bui, Keegan McCaffrey, Bree Barbeau, Andrea George, Carolyn Rose, Sarah Willardson, Amy Carter, Christopher Smoot, Allyn Nakashima, Angela Dunn

**Affiliations:** ^1^Utah Department of Health; ^2^Epidemic Intelligence Service, CDC ^3^Salt Lake County Health Department, Salt Lake City, Utah; ^4^Summit County Health Department, Coalville, Utah; ^5^Davis County Health Department, Clearfield, Utah; ^6^Weber-Morgan Health Department, Ogden, Utah; ^7^Wasatch County Health Department, Heber City, Utah.

Coronavirus disease 2019 (COVID-19) has had a substantial impact on racial and ethnic minority populations and essential workers in the United States, but the role of geographic social and economic inequities (i.e., deprivation) in these disparities has not been examined (*1*,*2*). As of July 9, 2020, Utah had reported 27,356 confirmed COVID-19 cases. To better understand how area-level deprivation might reinforce ethnic, racial, and workplace-based COVID-19 inequities (*3*), the Utah Department of Health (UDOH) analyzed confirmed cases of infection with SARS-CoV-2 (the virus that causes COVID-19), COVID-19 hospitalizations, and SARS-CoV-2 testing rates in relation to deprivation as measured by Utah’s Health Improvement Index (HII) (*4*). Age-weighted odds ratios (weighted ORs) were calculated by weighting rates for four age groups (≤24, 25–44, 45–64, and ≥65 years) to a 2000 U.S. Census age-standardized population. Odds of infection increased with level of deprivation and were two times greater in high-deprivation areas (weighted OR = 2.08; 95% confidence interval [CI] = 1.99–2.17) and three times greater (weighted OR = 3.11; 95% CI = 2.98–3.24) in very high-deprivation areas, compared with those in very low-deprivation areas. Odds of hospitalization and testing also increased with deprivation, but to a lesser extent. Local jurisdictions should use measures of deprivation and other social determinants of health to enhance transmission reduction strategies (e.g., increasing availability and accessibility of SARS-CoV-2 testing and distributing prevention guidance) to areas with greatest need. These strategies might include increasing availability and accessibility of SARS-CoV-2 testing, contact tracing, isolation options, preventive care, disease management, and prevention guidance to facilities (e.g., clinics, community centers, and businesses) in areas with high levels of deprivation.

Confirmed COVID-19 cases reported by local health departments and UDOH to the Utah National Electronic Disease Surveillance System during March 3–July 9, 2020, were included in the analysis. Addresses were used to assign cases to one of 99 Utah small statistical areas,* each with an HII score ranging from 72 to 160 (*4*). HII is a composite index calculated using nine indicators from the Utah Behavioral Risk Factor Surveillance System (BRFSS) (*5*): 1) median family income; 2) income disparity (a logarithmic ratio of households with <$10,000 income to ≥$50,000 income); 3) percentage of home ownership; 4) percentage of unemployment; 5) percentage of families below poverty threshold; 6) percentage of single-parent households with children aged <18 years; 7) percentage of population aged ≥25 years with <9 years of education; 8) percentage of population aged ≥25 years with at least a high school diploma; and 9) percentage of population at <150% of the poverty threshold (*6*). HII is categorized into quintiles: very low (least deprived), low, average, high, and very high (most deprived). Lower-deprivation areas are concentrated in many urban and suburban parts of northern Utah (e.g., Salt Lake, Davis, and Wasatch counties); higher-deprivation areas are generally in rural central and southern Utah, western Salt Lake City metropolitan area, and parts of other cities (e.g., Ogden and Logan).

COVID-19 incidence by HII quintile was calculated as the number of COVID-19 cases confirmed by real-time reverse transcription–polymerase chain reaction (RT-PCR) testing per 100,000 persons during March 3–July 9, 2020. Hospitalization rates were calculated as the number of patients with confirmed COVID-19 cases admitted to hospitals per 1,000 COVID-19 patients. SARS-CoV-2 testing rates were calculated as the number of persons whose specimens were tested by real-time RT-PCR at least once per 100,000 persons. Percent positivity was calculated as the percentage of persons who received a positive SARS-CoV-2 test result among those tested. Binary logistic regression was used to calculate unadjusted odds ratios (ORs) for incidence, hospitalization, and testing in each HII quintile, with the very low (least deprived) quintile as the referent. Rates for four age groups (≤24, 25–44, 25–64, and ≥65 years) were weighted to a U.S. Census 2000 age-standardized population to calculate age-weighted rates and weighted ORs. This activity was reviewed by CDC and was conducted consistent with applicable federal law and CDC policy.^†^

HII quintiles were further characterized by 10 variables. Four variables came from BRFSS, constituting percentages of residents who 1) identified as a race other than White (non-White), 2) identified as Hispanic or Latino (Hispanic); 3) were food insecure; or 4) were uninsured. Another six variables came from 2018 American Community Survey 5-year estimate data (*7*) on percentages of workers in high-risk sectors in Utah (*3*) including 1) food preparation and serving; 2) building and grounds cleaning and maintenance; 3) production; 4) construction and extraction; and 5) transportation and material moving; and 6) the percentage living in residences with one or more persons per room. SAS software (version 9.4; SAS Institute) was used for all analyses.

Incidence of confirmed COVID-19 increased with area deprivation level, from 545 in very low-deprivation areas to 674 in low-, 811 in average-, 1,124 in high-, and 1,674 in very high-deprivation areas (Table 1). Age-weighted incidences were slightly higher in all HII quintiles.

**TABLE 1:** Characteristics of COVID-19 outbreak, by quintiles of health improvement index areas — Utah, March 3–July 9, 2020

Characteristic	Level of deprivation				
	Very low (least deprived)	Low	Average	High	Very high (most deprived)
**Population in 2018**	**585,696**	**853,813**	**625,971**	**578,836**	**516,702**
**Cases**					
No. of cases	3,119	5,585	4,943	6,324	8,177
Incidence* (95% CI)	533 (514–552)	654 (637–6725)	790 (768–812)	1,093 (1,066–1,121)	1,583 (1,548–1,617)
Incidence,* weighted^†^ (95% CI)	545 (905–927)	674 (656–692)	811 (788–834)	1,124 (1,096–1,153)	1,674 (1,637–1,712)
**Hospitalization**					
No. of hospitalizations	160	324	346	375	576
Hospitalization rate^§^ (95% CI)	51 (44–60)	58 (52–65)	70 (63–78)	59 (53–66)	70 (65–76)
Hospitalization rate,^§^ weighted^†^ (95% CI)	51 (43–60)	62 (55–69)	76 (68–84)	69 (61–76)	81 (74–89)
**Testing**					
No. of persons tested for COVID-19	62,801	94,926	70,151	74,994	69,103
Testing rate^¶^ (95% CI)	10,723 (10,639–10,807)	11,118 (11,047–11,189)	11,207 (11,124–11,290)	12,956 (12,863–13,049)	13,374 (13,274–13,474)
Testing rate,^¶^ weighted^†^ (95% CI)	11,143 (11,055–11,230)	11,614 (11,540–11,690)	11,438 (11,353–11,523)	13,433 (13,336–13,530)	14,164 (14,056–14,271)
Percentage positive **	5.0	6.0	7.2	8.6	12.0

**Abbreviations:** CI = confidence interval; COVID-19 = coronavirus disease 2019.

* Cases per 100,000 population during March 3–July 9, 2020.

^†^ Calculated by weighting rates for four age groups (≤24, 25–44, 25–64, and ≥65 years) to a U.S. Census 2000 age-standardized population.

^§^ Hospitalizations per 1,000 cases.

^¶^ Determined by number of persons tested at least once per 100,000 persons.

** Percentage of persons who received a positive SARS-CoV-2 test result among all those tested.

Hospitalization rates were similar in very high- (70 per 1,000 cases) and average- (70) deprivation areas and <60 in very low- (51), low- (58), and high- (59) deprivation areas. Age-weighting resulted in higher hospitalization rates, especially in higher deprivation quintiles.

Testing rates also increased with deprivation level; the rate in very high-deprivation areas (13,374 per 100,000 persons) was approximately 25% higher than that in very low-deprivation areas (10,723). Age-weighted testing rates were higher than were unadjusted rates. Percentage test positivity increased with deprivation level, from 5.0% in very low-deprivation areas to 12.0% in very high areas.

Compared with persons living in very low-deprivation areas, age-weighted odds of having confirmed SARS-CoV-2 infection were significantly higher for persons in low- (weighted OR = 1.23), average- (1.49), high- (2.08), or very high- (3.11) deprivation areas (Table 2). Compared with patients living in very low-deprivation areas, the odds of hospitalization were significantly higher for those residing in low- (1.22), average- (1.52), high- (1.37), or very high- (1.64) deprivation areas. Odds of testing were similar among persons in low- (1.05) or average- (1.03) deprivation areas, compared with those in very low-deprivation areas, and were slightly higher among those in high- (1.23) and very high-deprivation areas (1.31).

**TABLE 2:** Unadjusted odds ratios (ORs), weighted* ORs, and 95% confidence intervals (CIs) for COVID-19 infection, hospitalization, and testing, by quintiles of health improvement index areas — Utah, March 3–July 9, 2020

Characteristic	Level of deprivation				
	Very low (least deprived)	Low	Average	High	Very high (most deprived)
**Cases**					
OR (95% CI) for confirmed SARS-CoV-2 infection	Referent	1.23 (1.17–1.29)	1.49 (1.42–1.56)	2.06 (1.98–2.15)	3.00 (2.88–3.13)
Weighted OR (95% CI) for confirmed SARS-CoV-2 infection	Referent	1.23 (1.19–1.29)	1.49 (1.43–1.56)	2.08 (1.99–2.17)	3.11 (2.98–3.24)
**Hospitalization**					
OR (95% CI) for hospitalization of a patient with a confirmed case	Referent	1.14 (0.94–1.38)	1.39 (1.15–1.69)	1.16 (0.96–1.41)	1.40 (1.17–1.68)
Weighted OR (95% CI) for hospitalization of a patient with a confirmed case	Referent	1.22 (1.00–1.47)	1.52 (1.26–1.84)	1.37 (1.14–1.65)	1.64 (1.38–1.97)
**Testing**					
OR (95% CI) for having been tested	Referent	1.04 (1.03–1.05)	1.05 (1.04–1.06)	1.24 (1.23–1.25)	1.29 (1.27–1.30)
Weighted OR for having been tested	Referent	1.05 (1.04–1.06)	1.03 (1.02–1.04)	1.23 (1.22–1.25)	1.31 (1.30–1.33)

**Abbreviation:** COVID-19 = coronavirus disease 2019.

* Weighted ORs were calculated by estimating the number of persons with the outcome of interest based on the age-weighted rates for each Health Improvement Index quintile. Confidence intervals that do not span 1.0 are considered significant

Area-level demographic and socioeconomic characteristics were also correlated with deprivation level (Table 3). The population percentages of Hispanic (22.5%) and non-White (22.0%) residents in very high-deprivation areas were more than four and three times as high as those in very low-deprivation areas (5.1% and 7.0%, respectively). The proportion of food-insecure residents living in very high-deprivation areas (22.6%) was approximately twice that of those in very low-deprivation areas (13.5%), as was the proportion employed in a higher-risk sector (35.9% versus 17.7%). Similarly, proportions of uninsured residents and those living in residences with more than one occupant per room were four times as high (16.9% and 6.6%, respectively) in very high-deprivation areas as they were in very low-deprivation areas (4.2% and 1.5%, respectively).

**TABLE 3:** Demographic, socioeconomic, and occupational characteristics, by quintiles of health improvement index areas — Utah, March 3–July 9, 2020

Characteristic	% of population				
	Level of deprivation				
	Very low (least deprived)	Low	Average	High	Very high (most deprived)
**Population in 2018**	**585,696**	**853,813**	**625,971**	**578,836**	**516,702**
**Demographic**					
Hispanic or Latino	5.1	9.9	10.4	13.5	22.5
Non-White	7.0	10.1	12.5	13.8	22.0
**Socioeconomic**					
Food insecure*	13.5	17.3	20.1	23.7	26.6
Uninsured	4.2	6.9	10.2	10.5	16.9
Living in residence with >1 occupant per room	1.5	2.6	3.3	3.9	6.6
**Occupational**					
Food preparation and serving related	3.4	4.7	4.9	5.8	6.7
Building and grounds cleaning and maintenance	2.1	2.6	3.9	3.7	5.7
Construction and extraction	3.4	4.6	6.4	5.8	6.9
Production	3.9	5.6	6.7	8.3	8.4
Transportation and material moving	4.9	6.0	7.9	7.6	8.2
Any of the above occupational categories	17.7	23.5	29.8	31.2	35.9

* Determined based on households in which the head indicated insufficient balanced meals, portion sizes, or food availability or participation in food assistance programs because of socioeconomic circumstances.

## Discussion

During March 3–July 9, 2020, odds of SARS-CoV-2 infection among residents living in areas of very high deprivation were three times higher than those of residents of areas of very low deprivation. The difference in incidence between residents of high- and very high-deprivation areas was as large as that between residents of very low- and high-deprivation areas, suggesting that extreme deprivation could compound transmission. Odds of hospitalization among residents of very high-deprivation areas were 1.6 times those among residents of very low-deprivation areas. Odds of testing varied less with deprivation than did incidence or hospitalization. Age-weighting generally amplified odds ratios, reflecting the younger age profile among persons in high-deprivation area populations (e.g., younger Hispanic and Pacific Islander families).

Area-level deprivation could exacerbate the ethnic and racial inequalities in COVID-19 morbidity and mortality observed in previous studies (*1*–*3*). A recent New York City study found that odds of infection were lower among pregnant women living in neighborhoods with higher median incomes and higher among women living in neighborhoods with more densely populated households (*8*). The unexpectedly high odds of hospitalization in average-deprivation areas could reflect more health care–seeking behavior and access to health insurance in those areas compared with more deprived areas (*1*,*9*). Risk factors for COVID-19 might cluster within geographic areas. For example, persons living in deprived areas might be both more likely to work in settings where they could become infected (*3*) and to live in higher-density settings where household members could become secondarily infected (*8*). They might also be unable to adhere to isolation protocols because of work requirements, higher-density dwellings, or lack of private transportation (*10*).

The findings in this report are subject to at least six limitations. First, incidence and testing could be underestimated because of presymptomatic transmission of SARS-CoV-2 and mild disease, which might be unrecognized. Second, HII is a composite measure of deprivation, and it is difficult to know which of its constituent factors are driving associations or the degree to which HII is a stronger predictor than individual factors. Third, Utah’s small areas might not match up precisely with communities; some areas might have a higher HII score because of a transient student population with lower incomes, whereas areas with overall low scores might still have clusters of underserved communities, such as American Indians. Fourth, characterization of HII quintiles was limited to area-level variables from BRFSS and American Community Survey; other potentially influential variables (e.g., overall dwelling density) were not available. Fifth, area-level population data are drawn from estimates before the pandemic, and pertinent measures such as unemployment might have changed more recently. Finally, occupational sectors included were not exhaustive of all high-risk work, and professions (e.g., frontline workers versus managers) might better characterize risk across deprivation areas.

Public health agencies should use social determinants of health such as deprivation to assess area-level COVID-19 disparities and implement interventions to address those disparities exacerbated by living and working conditions (*9*). These interventions might include increasing availability and accessibility of SARS-CoV-2 testing, contact tracing, isolation options, and preventive care and disease management in more deprived areas and distributing prevention guidance to facilities (e.g., clinics, community centers, and businesses) in these areas. In addition, public health agencies should prepare linguistically and culturally appropriate materials in the first language of ethnocultural communities at risk located in deprived areas and build partnerships with organizations that could facilitate outreach to those communities (*9*). Such place-focused strategies could constitute novel approaches to reducing the disproportionate incidence of COVID-19 in socioeconomically and materially disadvantaged communities.

**Summary**

**What is already known about this topic?**

COVID-19 has disproportionately affected socially disadvantaged groups.

**What is added by this report?**

During March 3–June 9, 2020, odds of SARS-CoV-2 infection in very high-deprivation areas of Utah were three times higher than those in very low-deprivation areas; rates of hospitalization and testing were also higher in higher-deprivation areas. These areas were characterized by larger proportions of Hispanic and non-White residents, persons working in manual, essential, and public-facing sectors, more crowded housing, and food and health care insecurity.

**What are the implications for public health practice?**

Enhanced mitigation strategies might include increasing availability and accessibility of SARS-CoV-2 testing, contact tracing, isolation options, preventive care, disease management, and prevention guidance in more deprived areas.

## Footnotes

* Utah’s small statistical areas are areas delineated by the state to facilitate community-level reporting for the smallest units with enough data to be reliable. https://ibis.health.utah.gov/ibisph-view/pdf/resource/Algorithm.pdf.

† 45 C.F.R. part 46, 21 C.F.R. part 56; 42 U.S.C. Sect. 241(d); 5 U.S.C. Sect. 552a; 44 U.S.C. Sect. 3501 et seq.
